# Feasibility of Peroral Pancreatoscopy Using the 9-Fr eyeMAX for Surgical Planning in Main-Duct and Mixed-Type Intraductal Papillary Mucinous Neoplasms

**DOI:** 10.3390/diagnostics16101443

**Published:** 2026-05-09

**Authors:** Haruo Miwa, Kuniyasu Irie, Tomoko Fujiyoshi, Nene Sakai, Ryo Soma, Kozue Shibasaki, Yugo Ishino, Shotaro Tsunoda, Yuto Matsuoka, Tomomi Hamaguchi, Kazuki Endo, Ritsuko Oishi, Yuichi Suzuki, Hiromi Tsuchiya, Akihiro Funaoka, Yoshimasa Suzuki, Satoshi Komiyama, Yoshihiro Goda, Manabu Morimoto, Shin Maeda

**Affiliations:** 1 Gastroenterological Center, Yokohama City University Medical Center, Yokohama 232-0024, Japan; fujiyoshi.tom.vl@yokohama-cu.ac.jp (T.F.); sakai.nen.fa@yokohama-cu.ac.jp (N.S.); soma.ryo.hs@yokohama-cu.ac.jp (R.S.); shibasaki.koz.ah@yokohama-cu.ac.jp (K.S.); ishino.yug.cy@yokohama-cu.ac.jp (Y.I.); tsunoda.sho.au@yokohama-cu.ac.jp (S.T.); endo.kaz.bd@yokohama-cu.ac.jp (K.E.); oishi.rit.dd@yokohama-cu.ac.jp (R.O.); suzuki.yui.ar@yokohama-cu.ac.jp (Y.S.); h_tsuchiya@yokohama-cu.ac.jp (H.T.); t206071f@yokohama-cu.ac.jp (A.F.); skomiyam@yokohama-cu.ac.jp (S.K.); morimoto.man.vx@yokohama-cu.ac.jp (M.M.); 2 Department of Gastroenterology, Yokohama City University Graduate School of Medicine, Yokohama 236-0004, Japan; smaeda@yokohama-cu.ac.jp; 3 Department of Gastroenterology, Yokohama City University Hospital, Yokohama 236-0004, Japan; k_irie@yokohama-cu.ac.jp (K.I.); matsuoka.yut.qk@yokohama-cu.ac.jp (Y.M.); hamaguchi.tom.rg@yokohama-cu.ac.jp (T.H.); y_suzuki@yokohama-cu.ac.jp (Y.S.); y_gouda@yokohama-cu.ac.jp (Y.G.)

**Keywords:** intraductal papillary mucinous neoplasm, peroral pancreatoscopy, main pancreatic duct, mural nodule, preoperative evaluation, digital cholangioscope

## Abstract

**Background/Objectives**: The role of peroral pancreatoscopy (POPS) in patients with main-duct and mixed-type intraductal papillary mucinous neoplasm (MD/mixed-IPMN) remains unclear. This retrospective multicenter case series aimed to evaluate the feasibility and safety of POPS using the 9-Fr eyeMAX and to describe additional intraductal findings, potential surgical impact, and biopsy–surgery concordance. **Methods**: Consecutive patients with MD/mixed-IPMN in whom POPS was attempted between May 2023 and September 2025 were retrospectively analyzed. Pre-POPS imaging findings, POPS findings, procedural outcomes, adverse events, surgical outcomes, and concordance between POPS-guided biopsy and surgical pathology were evaluated. **Results**: Among the 20 patients, 14 were men, and the median age was 74.5 years (range, 46–83 years). IPMNs were classified as main-duct type in five patients (25%) and mixed type in 15 (75%). On endoscopic ultrasonography, mural nodules were identified in 14 patients (70%), with a median diameter of 11 mm (range, 3–25 mm). Endoscopic papillary intervention was performed in 6 patients (30%). The technical success rate of POPS insertion was 95% (19/20). Mural nodules were detected by POPS in 17 patients (85%), and biopsy under POPS guidance was performed a median of 5 times (range, 1–10). The adequate tissue sampling rate was 86% (88/102). Mild post-ERCP pancreatitis occurred in 1 patient (5%). Twelve patients (60%) underwent surgery. The concordance rate between POPS-targeted biopsy and surgical pathology was 45% (5/11); however, R0 resection was achieved in 11 patients (92%). **Conclusions**: In this small retrospective case series, POPS using the 9-Fr eyeMAX was feasible and provided additional intraductal information in selected patients. However, its diagnostic role remains adjunctive because POPS-guided biopsy showed limited reliability.

## 1. Introduction

Intraductal papillary mucinous neoplasm (IPMN) is a relatively common pancreatic cystic neoplasm with malignant potential [1]. Long-term surveillance studies have shown that IPMN is associated with a 5-year cumulative incidence of progression to IPMN-derived invasive carcinoma of 1.9% [2]. Most IPMNs are classified as branch-duct type, whereas main-duct and mixed IPMNs (MD/mixed-IPMNs) are less common [3]. However, MD/mixed-IPMNs are generally considered to carry a higher risk of malignancy than branch-duct-type IPMNs [4,5,6]. Accordingly, the 2024 International Consensus Guidelines recommend surgical resection for surgically suitable patients with these lesions, particularly when high-risk stigmata (HRS) are present [1].

Despite this recommendation, the optimal extent of pancreatectomy for MD/mixed-IPMN is often difficult to determine preoperatively [7]. Some lesions show longitudinal intraductal spread along the main pancreatic duct (MPD) beyond the apparent border of the main lesion, extending from the pancreatic head to the body, or vice versa, which may necessitate extended resection or even total pancreatectomy [8]. Therefore, an accurate preoperative evaluation of mural nodules and intraductal tumor spread is essential to avoid undertreatment and overtreatment. Cross-sectional imaging modalities, such as computed tomography (CT) and magnetic resonance cholangiopancreatography (MRCP), are useful for the overall assessment of pancreatic morphology; however, they may be insufficient for detecting small mural nodules or defining the precise longitudinal extent of intraductal lesions [9]. Although endoscopic ultrasonography (EUS) provides superior spatial resolution and is valuable for identifying mural nodules, it cannot always assess the entire MPD continuously along its course, and small or subtle lesions may still be overlooked.

Peroral pancreatoscopy (POPS) allows direct visualization of the MPD and may therefore provide additional information for the preoperative evaluation of MD/mixed-IPMN. Several studies have reported the usefulness of POPS in detecting mural nodules, evaluating intraductal tumor spread, and obtaining targeted biopsy samples [10,11,12]. Nevertheless, POPS has not been widely adopted in routine practice, and clear recommendations regarding its role remain limited. One important concern is the risk of post-endoscopic retrograde cholangiopancreatography (ERCP) pancreatitis, which may be related to increased intraductal pressure caused by saline irrigation or direct contact between the scope and the pancreatic duct wall during pancreatoscopy [13]. In addition, limited maneuverability of conventional scopes within a narrow or tortuous MPD may result in unstable scope control and excessive contact with the ductal epithelium, particularly at angulated segments.

**Figure 1:**
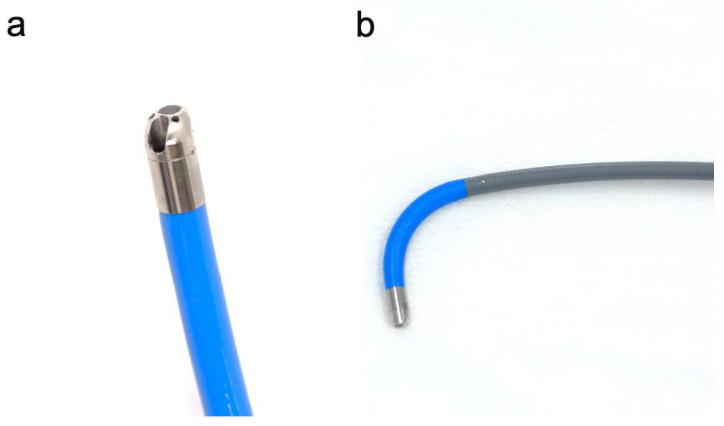
9-Fr eyeMAX (Micro-Tech, Nanjing, China). (**a**) The working channel and independent irrigation channel feature in a 3.2 mm body. (**b**) The highly maneuverable bending section facilitates smooth insertion into the pancreatic duct.

Recently, a novel slim cholangioscope (9-Fr eyeMAX; Micro-Tech, Nanjing, China) with an outer diameter of 3.2 mm, enhanced angulation capability with a 4-way tip deflection system, an independent irrigation channel, and high-quality imaging has been developed (Figure 1) [14]. These features may facilitate pancreatoscopic observation, even in slightly dilated or angled pancreatic ducts.

Therefore, this retrospective multicenter case series aimed to evaluate the feasibility and safety of POPS using the 9-Fr eyeMAX in patients with MD/mixed-IPMN and to describe its additional intraductal findings, potential impact on surgical planning, and concordance between POPS-guided biopsy and surgical pathology.

## 2. Materials and Methods

### 2.1. Study Design and Patients

This was a retrospective multicenter case series of consecutive patients with MD/mixed-IPMN in whom POPS using the 9-Fr eyeMAX was attempted between May 2023 and September 2025 at two tertiary referral centers. Patients were excluded if they had branch-duct-type IPMN alone, were aged <18 years, or declined the use of their clinical data through the institutional opt-out process. Written informed consent for the endoscopic procedure was obtained from all patients before POPS. Because of the retrospective study design, consent for the use of clinical data was obtained using an opt-out approach. This study was approved by the Institutional Review Board of Yokohama City University (approval number: F220300060; approval date: 23 January 2024) and was conducted in accordance with the Declaration of Helsinki (revised in Fortaleza, Brazil, October 2013).

### 2.2. Diagnosis of MD/Mixed-IPMN

The diagnosis of MD/mixed-IPMN was based on findings from cross-sectional imaging and endoscopic evaluation, including CT, MRCP, and EUS. Before POPS, CT was performed in 18 patients, MRCP in 16 patients, and EUS in all patients. The preoperative imaging protocol was not strictly standardized and was determined according to the clinical situation and institutional practice. MD-IPMN was defined as segmental or diffuse dilation of the MPD ≥ 5 mm without other causes of MPD obstruction [1]. In addition, patients with mural nodules within the MPD were included when the overall imaging findings were considered consistent with IPMN. Mixed IPMN is theoretically defined as IPMN involving both the main and branch ducts [1]; however, the distinction is often difficult to determine accurately based on preoperative imaging alone. Therefore, in this study, mixed-IPMN was defined as the presence of branch-duct cystic lesions in addition to the imaging features of MD-IPMN.

On MRCP, the maximum MPD diameter throughout the pancreas was measured to assess HRS. In addition, the minimal MPD diameter in the pancreatic head was measured to evaluate the feasibility of POPS insertion. The angle of the MPD between the pancreatic head and body was classified as acute (<90°) or mild (≥90°). On EUS, mural nodules were carefully assessed throughout the pancreas. Contrast-enhanced EUS were used to support the differentiation of mural nodules from mucus clots when available.

### 2.3. The 9-Fr eyeMAX

The eyeMAX series includes 11-Fr and 9-Fr models. In the present study, the 9-Fr eyeMAX was used for all procedures. This cholangiopancreatoscope has an outer diameter of 3.2 mm, a 1.1 mm working channel that allows the use of dedicated miniature biopsy forceps, and an independent irrigation channel for saline infusion. The scope is connected to a digital controller that provides high-quality endoscopic images. In addition, the 4-way tip deflection system improves maneuverability within the MPD and may facilitate advancement through angulated pancreatic duct anatomy.

### 2.4. POPS Procedure

This study included patients in whom insertion of the 9-Fr eyeMAX into the MPD was attempted for preoperative evaluation of MD/mixed-IPMN. All procedures were performed or directly supervised by expert endoscopists with substantial experience in more than 1000 ERCP procedures. POPS was performed using a dual-operator technique.

Patients were considered candidates for POPS when preoperative intraductal assessment was considered necessary to evaluate mural nodules and/or determine the longitudinal extent of the lesion for treatment planning. The primary procedural objective of POPS was to continuously observe the MPD from the head to the tail to assess intraductal tumor spread and support surgical decision making.

All procedures were performed under deep sedation using intravenous propofol, midazolam, and/or diazepam, with spontaneous respiration and continuous cardiorespiratory monitoring. A therapeutic duodenoscope (TJF-Q260V or TJF-Q290V; Olympus Medical Systems, Tokyo, Japan) was used for each procedure. After selective pancreatic duct cannulation, pancreatography was performed to evaluate ductal morphology. In patients without a fish-mouth papilla or with an inadequate papillary opening, endoscopic pancreatic sphincterotomy (EPST) or 6 mm endoscopic papillary balloon dilation (EPBD) was performed at the discretion of the endoscopist. Guidewire-assisted insertion was used as the standard approach. Non-guidewire insertion was considered only in selected patients with a sufficiently dilated pancreatic duct orifice and without marked MPD angulation to avoid intraductal mucosal erythema or erosion caused by guidewire contact.

After successful insertion into the MPD, the 9-Fr eyeMAX was advanced toward the pancreatic tail. The scope was then slowly withdrawn while carefully examining the ductal epithelium for mural nodules, papillary lesions, and other abnormalities. During observation, the minimum amount of saline required to maintain adequate visualization was manually injected through a syringe by the assistant. When visible mural nodules were identified, a targeted biopsy was performed using dedicated biopsy forceps. In selected cases, additional biopsy specimens were obtained from non-nodular areas to evaluate the extent of the tumor spread. After the procedure, a 5-Fr endoscopic nasopancreatic drainage tube was occasionally placed for serial pancreatic juice aspiration cytological examination, depending on the clinical situation and endoscopist’s judgment. All biopsy and surgical specimens were evaluated by experienced gastrointestinal pathologists at each institution.

### 2.5. Definitions and Outcomes

The primary outcome of this study was technical success, defined as the successful insertion of the 9-Fr eyeMAX into the MPD with completion of the intended pancreatoscopic observation.

Secondary outcomes included procedural findings, mural nodule detection, biopsy performance, adequate tissue sampling, adverse events, and concordance between POPS-guided biopsy and surgical pathology in resected cases. Adequate tissue sampling was defined as the successful acquisition of tissue specimens sufficient for pathological evaluation.

Adverse events were evaluated according to the American Society for Gastrointestinal Endoscopy lexicon and graded using the ASGE Severity Grading System [15]. Early adverse events were defined as those occurring within 14 days after the procedure. Post-ERCP pancreatitis was diagnosed according to established consensus criteria.

Owing to the small size and often insufficient nature of tissue samples obtained under POPS for reliable dysplasia grading, biopsy specimens were categorized as intraductal papillary mucinous carcinoma (IPMC), intraductal papillary mucinous adenoma (IPMA), or having no neoplastic tissue. In contrast, the final histopathological diagnosis of resected specimens was classified as low-grade dysplasia (LGD), high-grade dysplasia (HGD), or invasive carcinoma (IC) according to the International Consensus Guidelines [1]. HGD and IC were considered malignant.

Among patients who subsequently underwent surgical resection after successful POPS, concordance between POPS-guided biopsy and final surgical pathology was assessed. For this comparison, IPMA on POPS-guided biopsy was considered to correspond to LGD, whereas IPMC was considered to correspond to HGD or IC.

### 2.6. Statistical Analysis

All analyses were performed using JMP Pro (version 19; SAS Institute Inc., Cary, NC, USA). Categorical variables are expressed as numbers and percentages, and continuous variables are presented as median values with ranges. Technical success and adequate tissue sampling rates are presented with 95% confidence intervals. Concordance between POPS-guided biopsy and final surgical pathology was assessed descriptively among patients who underwent surgical resection after successful POPS. For this analysis, IPMA on POPS-guided biopsy was considered concordant with LGD in the resected specimen, whereas IPMC was considered concordant with HGD or IC. No formal hypothesis testing was performed, because of the small sample size and exploratory nature of this study.

## 3. Results

### 3.1. Patients’ Characteristics

The patients’ characteristics are summarized in Table 1. A total of 20 consecutive patients, including 14 men and 6 women, with a median age of 74.5 years (range, 46–83 years), underwent POPS using the 9-Fr eyeMAX. IPMNs were classified as main-duct type in five patients (25%) and mixed type in 15 (75%). On MRCP, the median maximum MPD diameter was 10 mm (range, 4–24 mm), and 11 (55%) patients had an MPD diameter ≥ 10 mm. The median minimal MPD diameter in the pancreatic head was 8 mm (range, 2–21 mm), whereas in four (20%) patients, it was <5 mm. Acute angulation of the MPD (<90°) between the pancreatic head and body was observed in nine (45%) patients. On EUS, mural nodules were identified in 14 (70%) patients, with a median maximum diameter of 11 mm (range, 3–25 mm). HRS was present in 16 (80%) patients, including enhancing mural nodules ≥ 5 mm in 12 (60%) and MPD dilation ≥10 mm in 11 (55%).

### 3.2. Procedural Outcomes of Peroral Pancreatoscopy

**Figure 2:**
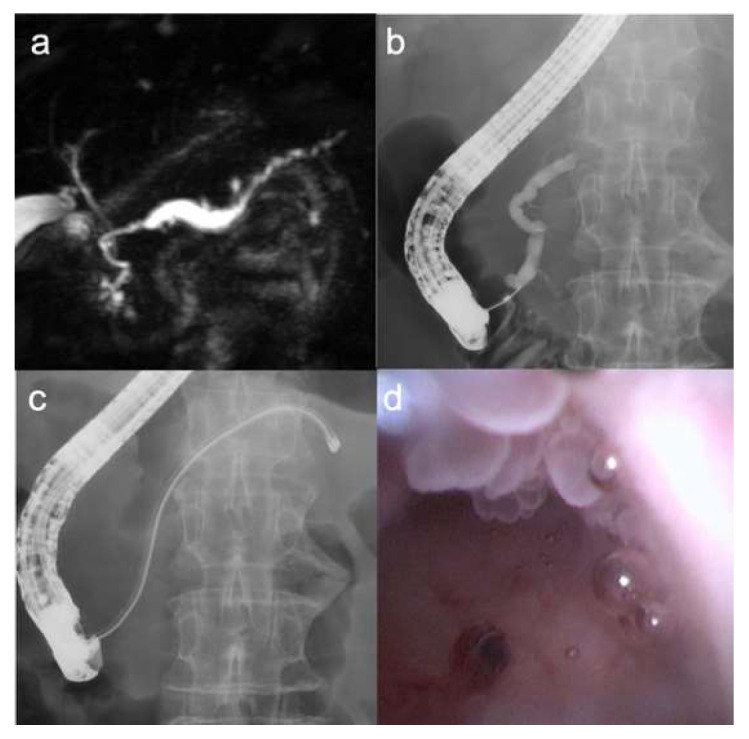
A patient with high-grade dysplasia in the pancreatic body. (**a**) Magnetic resonance cholangiopancreatography shows a thin main pancreatic duct in the pancreatic head. (**b**) Pancreatography shows an acute angulation of the main pancreatic duct in the pancreatic head. (**c**) The 9-Fr eyeMAX was successfully inserted into the pancreatic tail over the guidewire. (**d**) Mural nodules are identified in the pancreatic body. The biopsy specimen showed no neoplastic tissue because the biopsy forceps could not adequately sample the opposite side of the lesion.

**Figure 3:**
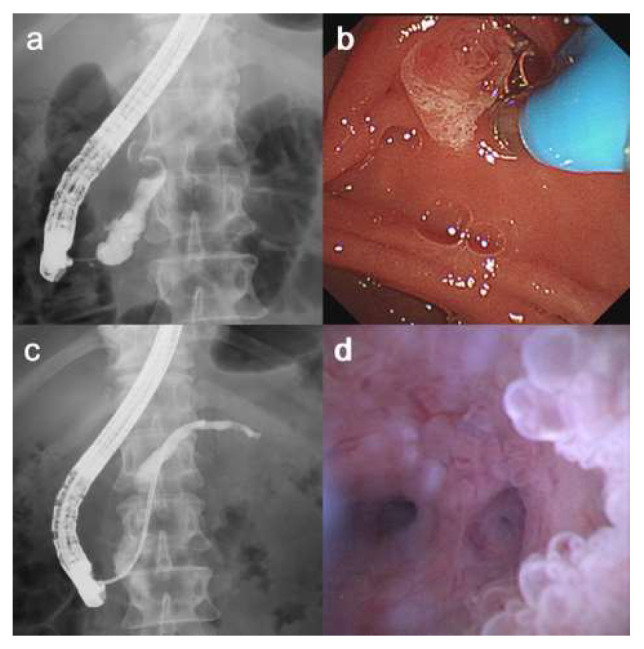
A patient with low-grade dysplasia in the pancreatic head. (**a**) Pancreatography shows dilation of the main pancreatic duct in the pancreatic head. (**b**) The 9-Fr eyeMAX is subsequently inserted into the main pancreatic duct without a guidewire. (**c**) The scope is advanced to the pancreatic tail. (**d**) Biopsy of the mural nodules in the pancreatic head revealed IPMA.

Details of the POPS are shown in Table 2. A fish-mouth sign of the papilla was observed in 16 patients (80%). Papillary intervention was required in 6 patients, including EPST in 2 and EPBD in 4. Technical success was achieved in 19 of 20 patients (95.0%; 95% CI, 75.1–99.9). Technical failure occurred in a patient with a minimal MPD diameter of 2 mm in the pancreatic head, in whom the 9-Fr eyeMAX could not be advanced through the papilla despite EPST. In the remaining technically successful cases, the scope could be advanced to the pancreatic tail. The high tip maneuverability of the eyeMAX and the dual-operator technique enabled advancement into the pancreatic body and tail even in cases with acute MPD angulation or an MPD diameter of <5 mm (Figure 2). In four patients (20%), the eyeMAX was inserted without guidewire assistance (Figure 3). Mural nodules were identified by POPS in 17 patients, including nodules located in the pancreatic head in 12 patients, in the body/tail in 3, and diffusely throughout the MPD in 2. Of the six patients without mural nodules on EUS, POPS detected them in five.

POPS-guided biopsy was performed in 16 patients, with a median of five biopsies (range, 1–10) per patient. Adequate tissue sampling was achieved in 88 of 102 biopsy specimens (86.3%; 95% CI, 78.0–92.3). Biopsy of mural nodules revealed IPMC in two patients and IPMA in 11, whereas no neoplastic tissue was obtained in two and the specimen was insufficient in one. Biopsy from non-nodular areas was performed in 16 patients and showed IPMA in two and no neoplastic tissue in 14. Nasopancreatic drainage was placed after the procedure in 13 patients (65%). Post-procedural asymptomatic hyperamylasemia occurred in one patient (5.0%). Regarding adverse events, only one patient (5.0%) developed mild post-ERCP pancreatitis, which resolved with conservative treatment, and no bleeding, perforation, or other adverse events were observed.

To clarify the incremental information obtained by POPS over standard imaging, a patient-level comparison of CT/MRCP, EUS, POPS findings, additional findings, and clinical impact is shown in Table 3. Compared with CT and MRCP, POPS more frequently allowed direct visualization of mural nodules. In contrast, the incremental detection of mural nodules over EUS was limited. Among the six patients without mural nodules on EUS, POPS newly identified mural nodules in five patients. In two patients whose mural nodules appeared localized to the pancreatic body and tail on EUS, POPS demonstrated diffuse mural nodules throughout the MPD. In these two patients, distal pancreatectomy was considered insufficient, whereas total pancreatectomy was considered excessively invasive; therefore, observation was selected after comprehensive assessment.

### 3.3. Surgical Outcomes

Of the 19 patients who successfully underwent POPS, 12 subsequently underwent surgical resection. Of the remaining seven patients, three did not have HRS, and four were managed conservatively based on patient preference, comorbidities, and comprehensive assessment. Surgical procedures included pancreatoduodenectomy in 10 patients and distal pancreatectomy in two, and the surgical margin was determined based on comprehensive preoperative assessment, including standard imaging and POPS findings when available. Details of the surgically treated patients are summarized in Table 4. Among the six patients without mural nodules on EUS, POPS newly identified them in five, and four of these patients subsequently underwent surgery based on comprehensive assessment, including POPS findings. In contrast, POPS failed to identify a mural nodule in one patient because the lesion was located within a branch-duct cyst.

The final pathological examination confirmed MD/mixed-IPMN in all resected cases and demonstrated IC in three patients, HGD in five, and LGD in four. R0 resection was achieved in 11 of the 12 patients, and LGD was present at the surgical margin in the remaining patient. Among the 12 patients who underwent surgical resection, concordance between POPS-guided biopsy and final surgical pathology was assessed in 11 patients who underwent both POPS-guided biopsy and surgery. Concordance was observed in five of these 11 patients (45%). In the remaining six patients, biopsy findings underestimated the final pathology, with HGD or IC identified in the resected specimens despite biopsy results of IPMA or no neoplastic tissue.

## 4. Discussion

This retrospective multicenter case series evaluated the feasibility, safety, and early clinical experience of POPS using the 9-Fr eyeMAX in selected patients with MD/mixed-IPMN. The technical success rate was high, and adverse events were limited in this cohort. POPS also provided additional intraductal information in selected patients, including detected mural nodules and more extensive intraductal involvement than suggested by EUS. However, the diagnostic concordance between POPS-guided biopsy and final surgical pathology was limited. Therefore, the present findings should be interpreted as preliminary and hypothesis-generating. POPS using the 9-Fr eyeMAX may serve as an adjunctive tool for selected patients in whom additional intraductal visualization is expected to assist surgical planning, rather than as a stand-alone diagnostic modality.

The utility of POPS for IPMN has been reported previously, including in systematic reviews and meta-analyses [11]. Earlier studies have shown that POPS may be useful for detecting mural nodules, evaluating intraductal tumor spread, and obtaining targeted biopsy specimens [16,17]. In some reports, pancreatoscopic findings have influenced surgical planning in 13–62% of patients [11]. However, these studies have also highlighted the limitations of conventional POPS, particularly the relatively high incidence of post-ERCP pancreatitis and the technical difficulty of advancing the scope through a narrow or tortuous MPD [17,18]. More recently, next-generation digital cholangioscopes, such as the SpyGlass DS system, have improved maneuverability and image quality through four-way tip deflection and independent irrigation [19,20]. Nevertheless, the outer diameter of such devices remains relatively large, which may still contribute to ductal trauma and impaired outflow during irrigation. Therefore, the feasibility and safety of POPS in challenging pancreatic duct anatomy remain important concerns.

The 9-Fr eyeMAX may address some of the technical limitations of conventional pancreatoscopy. Its smaller outer diameter and reduced cross-sectional area may help preserve the space between the scope and ductal wall and maintain the outflow of saline and pancreatic juice during the procedure [14]. In addition, its four-way tip deflection system may also facilitate advancement through angulated segments of the MPD [21]. Improved digital imaging and the independent irrigation channel may also allow adequate visualization with minimal saline irrigation. In the present study, these characteristics appeared to contribute to the high technical success rate, including successful insertion in patients with an MPD diameter of 3–4 mm. However, these observations should be interpreted cautiously because this study was not designed to demonstrate superiority over other pancreatoscopy systems.

Preoperative POPS should be regarded as an adjunctive tool rather than a modality for determining the indication for surgery itself [1,11]. Established imaging findings, particularly those detected by EUS and cross-sectional imaging, remain central to the identification of surgical candidates [22]. POPS appears to be most useful in selected patients who are already being considered for surgical treatment. In such cases, direct visualization of the MPD may provide additional information regarding the presence of subtle mural nodules and the longitudinal extent of intraductal tumor spread [11,12,16,17]. This is clinically relevant because the extent of pancreatectomy in MD/mixed-IPMN can be difficult to determine preoperatively, especially when the lesion extends beyond the apparent border of the main lesion along the MPD. The potential benefit of POPS should be considered in the context of surgical planning rather than definitive histological diagnosis. In selected patients, direct visualization of subtle mural nodules or longitudinal intraductal spread may help avoid under-surgery, such as insufficient PD or DP when disease extent is underestimated, as well as over-surgery, such as unnecessary total pancreatectomy when disease extent is overestimated. However, this potential benefit remains exploratory in the present study and requires validation in larger prospective cohorts.

In the present study, adverse events were limited, with only one case of mild post-ERCP pancreatitis and one case of asymptomatic hyperamylasemia. One possible explanation is the slim profile of the device, which may reduce ductal obstruction and facilitate drainage during irrigation. Another possible factor is that, unlike biliary endoscopy, pancreatoscopy often requires only minimal saline irrigation because pancreatic juice is transparent, and visualization can frequently be maintained without filling the duct. However, POPS is an invasive ERCP-based procedure that carries potential risks, including post-ERCP pancreatitis and complications related to pancreatic duct instrumentation and irrigation. Therefore, the low rate of pancreatitis observed in this study should be interpreted cautiously because of the small sample size, careful patient selection, expert operators, and limited irrigation. POPS should be reserved for selected patients in whom additional intraductal information is expected to influence management or surgical planning.

**Figure 4:**
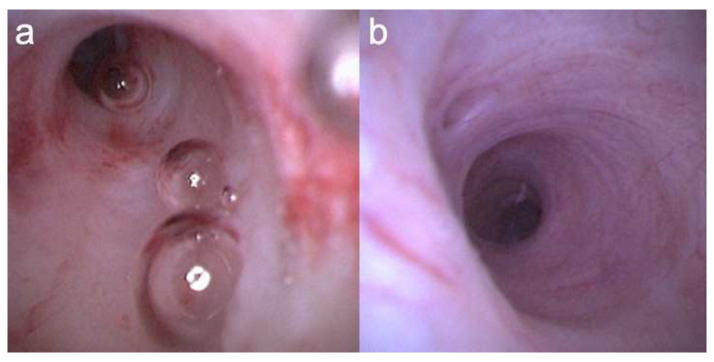
Comparison of pancreatoscopy findings with and without guidewire assistance. (**a**) With guidewire assistance, focal mucosal erythema is observed, possibly due to guidewire contact. (**b**) In a selected case without guidewire assistance, no obvious guidewire-related mucosal erythema is observed.

In selected cases with a markedly dilated MPD and a sufficiently open pancreatic orifice, POPS was performed without guidewire assistance to avoid mucosal erythema or erosion caused by guidewire contact (Figure 4). This approach may improve visualization of subtle mucosal findings; however, its safety, reproducibility, and clinical relevance require further evaluation.

The low concordance rate between POPS-guided biopsy and surgical pathology is an important limitation of this study. Among patients who underwent both POPS-guided biopsy and surgical resection, concordance was observed in only 5 of 11 patients. POPS-guided biopsy underestimated the final pathological diagnosis in several patients. This may be related to the small size of biopsy specimens, technical difficulty in sampling some mural nodules, the marked histological heterogeneity of IPMN, and the possibility that the most dysplastic component may not always be sampled [1,23]. Therefore, POPS-guided biopsy should not currently be regarded as a reliable stand-alone method for grading dysplasia or excluding HGD/IC. Biopsy results should be interpreted as supportive information together with EUS, MRCP, CT, direct pancreatoscopic findings, and clinical context.

This study has several limitations. First, the sample size was small, and only 12 patients underwent surgical resection; therefore, the diagnostic and clinical impact of POPS could not be evaluated with sufficient statistical power. Second, the retrospective design and inclusion of selected patients who were considered to require intraductal assessment may have introduced selection bias. Third, the preoperative imaging protocol and procedural strategy were not strictly standardized between centers or patients, including the use of contrast-enhanced EUS, EPST or EPBD, guidewire-assisted or non-guidewire insertion, irrigation volume, biopsy strategy, and nasopancreatic drainage placement. Fourth, this study did not include a direct comparison with other pancreatoscopy systems or with standard imaging alone. Fifth, no formal diagnostic-performance analysis was performed because of the small number of surgical cases and the exploratory nature of the study. Sixth, all procedures were performed or supervised by expert ERCP endoscopists at tertiary referral centers, which limits the generalizability of the findings to centers with less experience. Finally, the concordance between POPS-guided biopsy and surgical pathology was limited (5/11), and biopsy findings should not be used as a stand-alone basis for grading dysplasia or excluding HGD/IC.

## 5. Conclusions

Although POPS using the 9-Fr eyeMAX was feasible and provided additional intraductal information in selected patients, the concordance between POPS-guided biopsy and surgical pathology was limited. POPS-guided biopsy should not be used as a stand-alone method for grading dysplasia or excluding HGD/IC. Larger prospective studies with direct comparison to standard imaging are warranted to clarify the clinical role of POPS in surgical planning.

## Figures and Tables

**Table 1:** Patient characteristics.

Total number of patients	20
Age, years (range)	74.5 (46–83)
Sex	
Male	14 (70%)
Female	6 (30%)
IPMN subtype	
Main-duct type	5 (25%)
Mixed type	15 (75%)
Serum amylase, U/L (range)	82 (30–387)
Elevated serum amylase, *n* (%)	5 (25%)
Maximum diameter of MPD, mm (range)	10 (4–24)
<10 mm	9 (45%)
≥10 mm	11 (55%)
Minimal diameter of MPD in the pancreatic head, mm (range)	8 (2–21)
<5 mm	4 (20%)
5–10 mm	11 (55%)
>10 mm	5 (25%)
Angulation of MPD	
Acute (<90°)	9 (45%)
Mild (≥90°)	11 (55%)
Mural nodules detected on EUS	
Present	14 (70%)
Maximum diameter, mm (range)	11 (3–25)
High-risk stigmata	16 (80%)
Enhancing nodule ≥ 5 mm	12 (60%)
MPD ≥ 10 mm	11 (55%)
Obstructive jaundice	0 (0%)

Data  are presented as medians (range) or *n* (%). IPMN, intraductal papillary mucinous neoplasm; MPD, main pancreatic duct; EUS, endoscopic ultrasonography.

**Table 2:** Details of peroral pancreatoscopy.

Technical success, % (95% CI)	95.0 (75.1–99.9)
Procedure time, min (range)	65 (48–106)
Fish-mouth sign of the papilla	16 (80%)
EPST	2 (10%)
EPBD	4 (20%)
POPS without guidewire assistance, *n* (%)	4 (20%)
Mural nodules detected on POPS, *n* (%)	17 (85%)
Biopsy under POPS, *n* (%)	16 (80%)
Median number of biopsies, *n* (range)	5 (1–10)
Rate of adequate tissue sampling, % (95% CI)	86.3 (78.0–92.3)
Total number of biopsies, *n*	102
Adequate specimens, *n*	88
Insufficient specimens, *n*	14
Biopsy from mural nodules	16 (80%)
IPMC	2
IPMA	11
No neoplastic tissue	2
Insufficient specimen	1
Biopsy from non-nodular area	16 (80%)
IPMA	2
No neoplastic tissue	14
Insufficient specimen	0
Nasopancreatic drainage, *n* (%)	13 (65%)
Asymptomatic hyperamylasemia, *n* (%)	1 (5.0%)
Adverse events, *n* (%)	
Pancreatitis	1 (50%)
Bleeding	0 (0%)
Perforation	0 (0%)
Others	0 (0%)

Abbreviations: EPST, endoscopic pancreatic sphincterotomy; EPBD, endoscopic papillary balloon dilation; 95% CIs, 95% confidence intervals; IPMC, intraductal papillary mucinous carcinoma; IPMA, intraductal papillary mucinous adenoma.

**Table 3:** Patient-level comparison of imaging findings, POPS findings, and clinical impact.

Case	CECT/MRCP MN	EUS MN	POPS MN	Additional Information from POPS	Treatment/Potential Surgical Impact
1	Absent/Absent	Ph	Ph	No additional MN information; concordant with EUS	PD; Ph-limited disease supported
2	Ph/Absent	Ph	Ph	No additional MN information; concordant with EUS	PD; Ph-limited disease supported
3	Absent/Absent	Ph	Ph	No additional MN information; concordant with EUS	PD; Ph-limited disease supported
4	Absent/Absent	Ph	Ph	No additional MN information; concordant with EUS	PD; Ph-limited disease supported
5	Absent/Absent	Absent	Ph	New Ph MN detected by POPS	PD; surgery supported after comprehensive assessment
6	Ph/Ph	Absent	Ph	POPS confirmed Ph MN suggested by CECT/MRCP despite negative EUS	PD; Ph-limited disease supported
7	Absent/Absent	Absent	Ph	New Ph MN detected by POPS	PD; surgery supported after comprehensive assessment
8	NP/Absent	Ph	Ph	No additional MN information; concordant with EUS	Observation; no surgical impact
9	Absent/NP	Absent	Ph	New Ph MN detected by POPS	PD; surgery supported after comprehensive assessment
10	Ph/NP	Ph	Absent	No additional MN information; MN not identified by POPS	PD; surgery based on standard imaging/EUS
11	Absent/Absent	Ph	Ph	No additional MN information; concordant with EUS	Observation; no surgical impact
12	Absent/Absent	Pbt	Pbt	No additional MN information; concordant with EUS	DP; Pbt-limited disease supported
13	Ph/NP	Ph	Failed	No additional information, due to technical failure	PD; surgery based on standard imaging/EUS
14	Pbt/NP	Pbt	Pbt	No additional MN information; concordant with EUS	DP; Pbt-limited disease supported
15	Absent/Absent	Pbt	Diffuse	More extensive MN distribution than EUS	Observation; DP considered insufficient, whereas TP was considered too invasive
16	NP/Absent	Absent	Ph	New Ph MN detected by POPS	Observation after comprehensive assessment
17	NP/Absent	Pbt	Diffuse	More extensive MN distribution than EUS	Observation; DP considered insufficient, whereas TP was considered too invasive
18	Absent/Absent	Ph	Ph	No additional MN information; concordant with EUS	PD; Ph-limited disease supported
19	Absent/Absent	Absent	Absent	No additional MN information	Observation; no surgical impact
20	Absent/Absent	Pbt	Pbt	No additional MN information; concordant with EUS	Observation after comprehensive assessment

MN, mural nodule; CECT, contrast-enhanced computed tomography; MRCP, magnetic resonance cholangiopancreatography; EUS, endoscopic ultrasonography; POPS, peroral pancreatoscopy; Ph, pancreatic head; Pbt, pancreatic body and tail; PD, pancreatoduodenectomy; DP, distal pancreatectomy; TP, total pancreatectomy; NP, not performed. Absent indicates that mural nodules were not detected. Diffuse indicates mural nodules extending throughout the main pancreatic duct. Treatment decisions were based on comprehensive assessment and not on POPS findings alone.

**Table 4:** Surgical cases with EUS, POPS, and histopathologic findings.

Case	Age	Sex	IPMN Type	MPD mm	EUS MN, mm	POPS MN	Biopsy from Mural Nodule	Biopsy from Non-Nodular Area	Surgery	Final Pathology	R0 Resection
1	54	F	Mixed	8	4	Present	No neoplastic	No neoplastic	PD	IC	Y
2	72	M	Mixed	16	11	Present	IPMC	No neoplastic	PD	IC	Y
3	56	M	Mixed	8	15	Present	IPMA	No neoplastic	PD	LGD	Y
4	69	M	MD	11	25	Present	IPMA	No neoplastic	PD	IC	Y
5	74	F	MD	10	Absent	Present	No neoplastic	No neoplastic	DP	HGD	Y
6	77	F	Mixed	14	Absent	Present	No neoplastic	No neoplastic	PD	HGD	Y
7	76	M	Mixed	14	Absent	Present	IPMA	No neoplastic	PD	LGD	Y
9	46	M	Mixed	10	Absent	Present	IPMA	IPMA	PD	LGD	Y
10	75	M	Mixed	18	14	Absent	Not performed	No neoplastic	PD	HGD	Y
12	80	M	MD	24	23	Present	IPMA	IPMA	DP	HGD	N
14	78	M	Mixed	9	16	Present	IPMA	No neoplastic	PD	HGD	Y
18	60	M	Mixed	14	5	Present	IPMA	Not performed	PD	LGD	Y

MPD, main pancreatic duct; EUS, endoscopic ultrasonography; POPS, peroral pancreatoscopy; MD, main-duct type; PD, pancreatoduodenectomy; DP, distal pancreatectomy; IC, invasive carcinoma; HGD, high-grade dysplasia; LGD, low-grade dysplasia; IPMC, intraductal papillary mucinous carcinoma; IPMA, intraductal papillary mucinous adenoma. No neoplastic indicates no neoplastic tissue in the biopsy specimen.

## Data Availability

Supporting data for the reported results are available from the corresponding author upon reasonable request. The data are not publicly available because of privacy restrictions.
